# FitSearch: a robust way to interpret a yeast fitness profile in terms of drug's mode-of-action

**DOI:** 10.1186/1471-2164-14-S1-S6

**Published:** 2013-01-21

**Authors:** Minho Lee, Sangjo Han, Hyeshik Chang, Youn-Sig Kwak, David M Weller, Dongsup Kim

**Affiliations:** 1 Department of Bio and Brain Engineering, Korea Advanced Institute of Science and Technology, 291 Daehak-ro, Yuseong-Gu, Daejeon, 305-701, Republic of Korea; 2 Bioinformatics Lab, Healthcare group, SK telecom, 9-1, Sunae-dong, Pundang-gu, Sungnam-si, Kyunggi-do, 463-784, Republic of Korea; 3 Department of Biological Science, Seoul National University, 599 Gwanakro, Gwanak-gu, Seoul, 151-747, Republic of Korea; 4 Department of Applied Biology, Gyeongsang National University, 501 Jinju-daero, Jinju, Gyeongnam, 660-701, Republic of Korea; 5 USDA-ARS, Root Disease and Biological Control Research Unit, 367 Johnson Hall, Washington State University, Pullman, Washington 99164-6430, USA

## Abstract

**Background:**

Yeast deletion-mutant collections have been successfully used to infer the mode-of-action of drugs especially by profiling chemical-genetic and genetic-genetic interactions on a genome-wide scale. Although tens of thousands of those profiles are publicly available, a lack of an accurate method for mining such data has been a major bottleneck for more widespread use of these useful resources.

**Results:**

For general usage of those public resources, we designed FitRankDB as a general repository of fitness profiles, and developed a new search algorithm, FitSearch, for identifying the profiles that have a high similarity score with statistical significance for a given fitness profile. We demonstrated that our new repository and algorithm are highly beneficial to researchers who attempting to make hypotheses based on unknown modes-of-action of bioactive compounds, regardless of the types of experiments that have been performed using yeast deletion-mutant collection in various types of different measurement platforms, especially non-chip-based platforms.

**Conclusions:**

We showed that our new database and algorithm are useful when attempting to construct a hypothesis regarding the unknown function of a bioactive compound through small-scale experiments with a yeast deletion collection in a platform independent manner. The FitRankDB and FitSearch enhance the ease of searching public yeast fitness profiles and obtaining insights into unknown mechanisms of action of drugs. FitSearch is freely available at http://fitsearch.kaist.ac.kr.

## Background

The collection of yeast deletion strains has been a powerful tool for systematic genome-wide functional analysis. A budding yeast deletion-mutant library has been available for more than ten years [1], and recently a fission yeast collection has also become available [2]. In particular, strain-specific molecular barcodes allow quantitative functional profiling of pooled deletion strains by using a TAG oligonucleotide microarray [3]. Among many types of functional profiles, the chemical-genetic profiles express quantitative values of growth defects of deletion strains in the presence of certain chemicals. The compendium of chemical-genetic profiles of heterozygous and homozygous deletion strains for a large number of chemicals has been successfully used to identify direct target proteins of drugs [4,5] as well as exploring their modes-of-action [6,7]. Such profile data can also be a valuable resource for many other applications in chemical genomics. In *S. cerevisiae*, thousands of chemical-genetic profiles have been generated so far and are publicly available [8]. Fitness data have been deposited in a recently developed public database called fitDB [8]; this database, however, only provides limited tools such as an online-interface for searching with keywords such as yeast ORFs or drug names.

In a large-scale study using a single measurement platform, researchers can easily compare fitness profiles using several well-known similarity measures. Then, by clustering the profiles, they can group bioactive compounds with a similar mode-of-action and make a plausible hypothesis about the unknown mode-of-action of a drug [7]. However, chemical-genetic profiles can be generated using many different measurement platforms such as DNA chip-based parallel measurements [4,5,7,9], high-density colony measurement on agar plates [10], high-density well plate-based optical density [11] or fluorescence [12] measurements in liquid culture. Moreover, the fitness scores can be expressed in many different ways (i.e., fold-ratio, z-score, p-value, ranks, or binary values expressing growth defects). Such difficulties complicate the process of relating one profile to another. This problem becomes more severe when only a limited number of yeast fitness profiles for a drug of interest are available, and more so when such profiles have been generated by non-chip-based measurement platforms. In such cases, it is very difficult to perform data-mining against the tens of thousands of public fitness profiles that may contain valuable information on the mode-of-action of the drug of interest.

**Figure 1:**
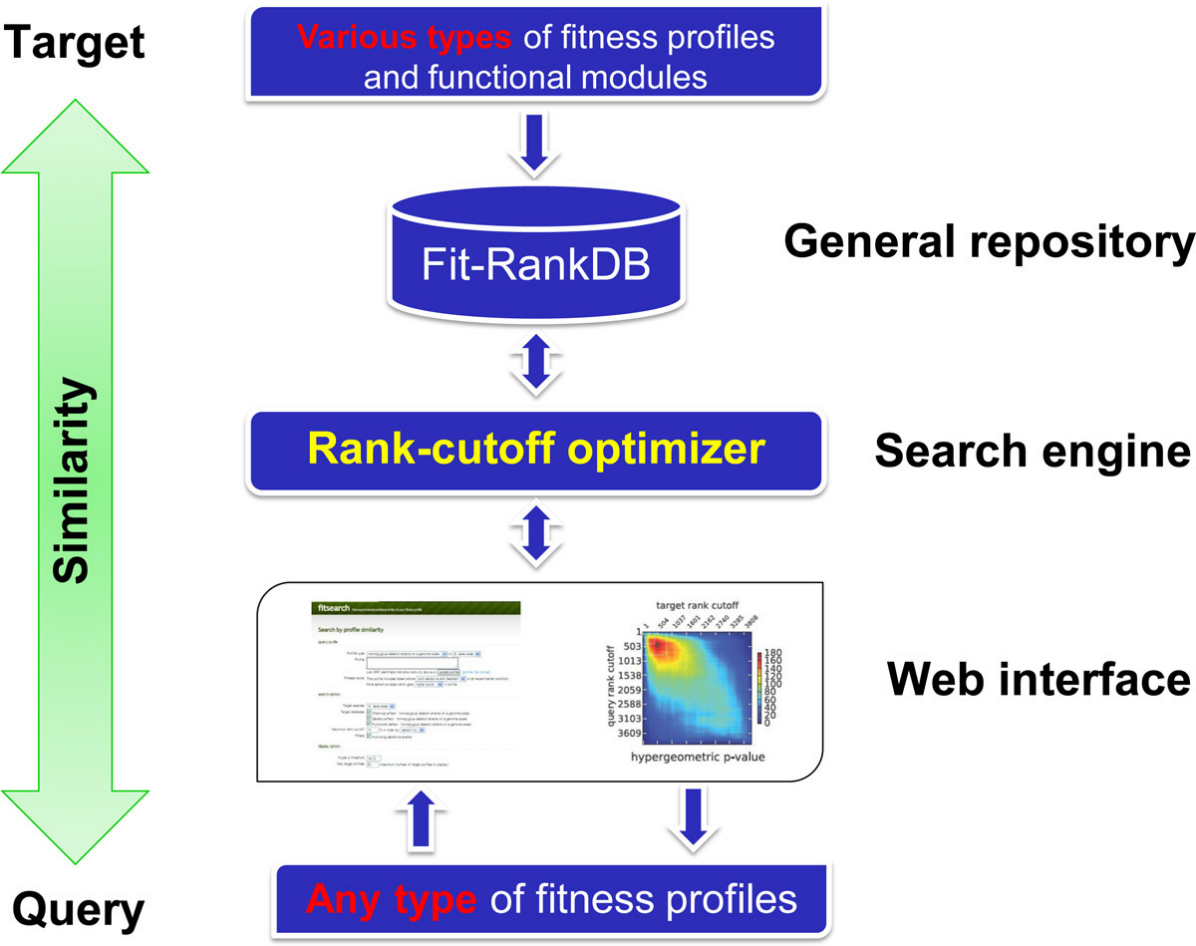
**Overall scheme of FitSearch**. Although researchers have only one or two yeast fitness profiles to their drugs of interest that have unknown toxicity mechanisms, they can easily perform data-mining against tens of thousands of public fitness profiles in order to obtain insight into the mechanism through the FitSearch website (http://fitsearch.kaist.ac.kr). When any type of yeast fitness profile is submitted as a query in the website, a similarity search to other public resources is performed by rank-cutoff optimizer through the FitSearch engine, which is a newly developed method using rank-based overlapping statistics (see the details in the Methods). Since available public resources are deposited in FitRankDB as a general repository for the FitSearch engine (see the details in the Methods), the similarity search can be performed more efficiently, thoroughly, and rapidly in the FitSearch website. Finally, users scrutinize characteristics of a list of drugs similar to their drug of interest and obtain clues or plausible hypotheses, which could also help them to design further bioassays.

In this study, we developed FitRankDB as a general repository of fitness profiles, and FitSearch as a new fitness similarity search algorithm which compares fitness profiles and calculates their similarity score and the corresponding statistical significance, regardless of the types of experimental setup by which they have been generated (Figure 1). The FitRankDB and FitSearch web service provides an uncomplicated means for searching tens of thousands of public yeast fitness profiles and obtaining insight into unknown modes-of-action of drugs.

## Methods

The ultimate objective of FitSearch is to provide a computational tool for interpreting any type of yeast fitness profiles in terms of the mode-of-action of a drug through comparison of various types of yeast fitness profiles and publicly available functional annotations. To do this, we first needed to create a general repository of yeast fitness profiles combined with genetic interaction information. Next, and more importantly, we needed a universal similarity measure for comparing profiles in a biologically meaningful way. Finally, for efficient and wider use of the resources, it was necessary to develop an easy-to-use public server in which users can choose and run several web applications, depending on their specific needs.

### FitRankDB, a general repository of yeast fitness profiles

Genome-wide yeast fitness profiles, using a yeast deletion library, have been used to infer the mode-of-action of a drug, genetic interactions, such as synthetic lethality, and functional annotation of unknown genes. We categorized those fitness profiles according to a yeast deletion library and a treatment, and collected them from public resources (Table 1). To make our collection a general repository for yeast fitness profiles, we defined a flexible standard file format using YAML (YAML Ain't Markup Language) to express all relevant information available. This yeast fitness YAML (called FitYAML) is a very simple format, which consists of three-letter keywords and their corresponding values. Currently, 32 three-letter keywords are defined, but this definition is scalable (see keyword definitions and download FitYAML at the website). Our internal curation system automatically extracts ranks of raw fitness scores and experimental information from FitYAML, and stores them into FitRankDB. For efficient computation, FitRankDB has two types of databases, Berkeley DB for rank information, and MySQL for experimental information. In addition, we collected various functional module definitions, such as protein complexes, to assist biological interpretation of fitness profiles into FitRankDB.

**Table 1:** Different types of yeast fitness profiles deposited in FitRankDB.

Type of treatment	Type of genome-wide deletion library	Type of fitness profile	Profile #
Chemical effect^1^	Homozygous deletion strains^3^	Chemical-genetic (Hom)	918
Chemical effect	Heterozygous deletion strains^3^	Chemical-genetic (Het)	1,530
Genetic effect^2^	Homozygous deletion strains	Genetic-genetic (Hom)	12,419

See the details at http://pombe.kaist.ac.kr/fitsearch/statistics/

^1 ^For example, drug, bioactive compounds or natural crude extracts

^2 ^For example, knock-out, over-expression or mutation of a gene

^3 ^Homozygous (or haploid) and heterozygous (or diploid) deletion collections of *S. cerevisiae *and *S. pombe *are commercially available at Open Biosystems (http://www.openbiosystems.com) and Bioneer (http://pombe.bioneer.co.kr), respectively.

Biogrid is a manually curated database, which provides various types of genetic interactions. It also contains information that enables us to distinguish query genes from array genes in a genetic interaction assay. To construct genetic-genetic profiles from the biogrid genetic interaction data, we extracted only the synthetic lethality (SL), synthetic growth defect (SD) and phenotypic enhancement (PE) datasets in *S. cerevisiae*, and ranked the array genes corresponding to each query gene according to the degree of growth defect: SL > SD > PE. In this procedure, array genes against the same query gene reported in different papers are merged (named 'Biogrid merged') or are separately deposited (named 'Biogrid individual') in FitRankDB. In addition, array genes against a query gene are assumed to be genes of strains with significant growth defects determined by genome-scale screening, and the query gene considered to be identified from a genetic treatment, such as gene knockout. Other large genetic interaction datasets from Epistatic Mini Array Profiles (E-MAP), were downloaded at http://interactome-cmp.ucsf.edu and deposited into FitRankDB in a similar way: 'chromosome function E-MAP' [13], 'signaling E-MAP' [14], 'early secretory pathway E-MAP' [15] and 'RNA processing E-MAP' [16].

### FitSearch, a rank cutoff optimizer as a search engine

Suppose that we are investigating whether two chemicals share a similar mode-of-action. One way to do this is to measure the similarity between the two fitness profiles of chemicals that may have been generated from different measurement platforms. Then, a requisite property for a new similarity measure is that the more similar the modes-of-action of the two chemicals are, the greater the similarity score should be. Realizing that the main difficulty in developing a biologically meaningful similarity measure arises from the fact that the profiles may have been generated from different types of experimental setup and that their fitness values may have been expressed in different ways, we first transformed the fitness values of each strain into their ranks. Next, considering that only the highly ranked strains that are significantly affected by a given chemical treatment are informative for inferring its mode-of-action, we chose the rank cutoff values for the two fitness profiles. Unlike previous methods where cutoff values were chosen rather arbitrarily, we developed an efficient dynamic programming algorithm to choose the two optimal rank cutoff values.

This algorithm named "rank cutoff optimizer" finds the optimal rank cutoffs by maximizing the statistical significance for overlap of the same set of strains in the two lists. The probability of overlapping by chance is known to follow the hyper-geometric distribution [17]. Suppose that there are *m_ij _*strains that co-occur in both the query profile, with the rank cutoff *i*, having *q_i _*strains and the target profile, with the rank cutoff *j*, having *t_j _*strains. Then the probability of such co-occurrence happening by random chance, *p*(*m_ij_*), is given by

(1) $$p\left(m_{ij}\right)=\frac{C\left(q_{i},m_{ij}\right)C\left(n-q_{i},t_{j}-m_{ij}\right)}{C\left(n,t_{j}\right)}$$

where *n *is the total number of strains, and *C*(*n, m*) is the number of ways of choosing *m *strains out of *n *strains. Of particular interest is the cumulative probability of *p*(*m*),

(2) $$Hp\left(m_{ij},q_{i},t_{j};n\right)=\overset{\text{min}\left(q_{i},t_{j}\right)}{\underset{m=m_{ij}}{\sum }}p\left(m\right)$$

which is simply the *p-value*. In addition, a p-value considering multiple hypothesis correction is defined using Bonferroni correction, which is a stringent correction method, given by

(3) $$\tilde{p}=Np$$

where *N *is represents the number of tests. This correction eventually increases the p-value. The rank cutoff optimizer calculates the optimal rank cutoffs, i* and j*, by minimizing the p-value, *i.e*.,

(4) $$\left(i^{*},j^{*}\right)=\underset{i,j=1..k}{\text{arg}\;\text{min}}\;Hp\left(m_{ij},q_{i},t_{j};n\right)$$

and reports both the overlapping significance defined as -log_10_(*p-value*) and the overlapping score, which is the Tanimoto coefficient given by

(5) $$T=\frac{m_{i^{*}j^{*}}}{q_{i^{*}}+t_{j^{*}}-m_{i^{*}j^{*}}}$$

**Figure 2:**
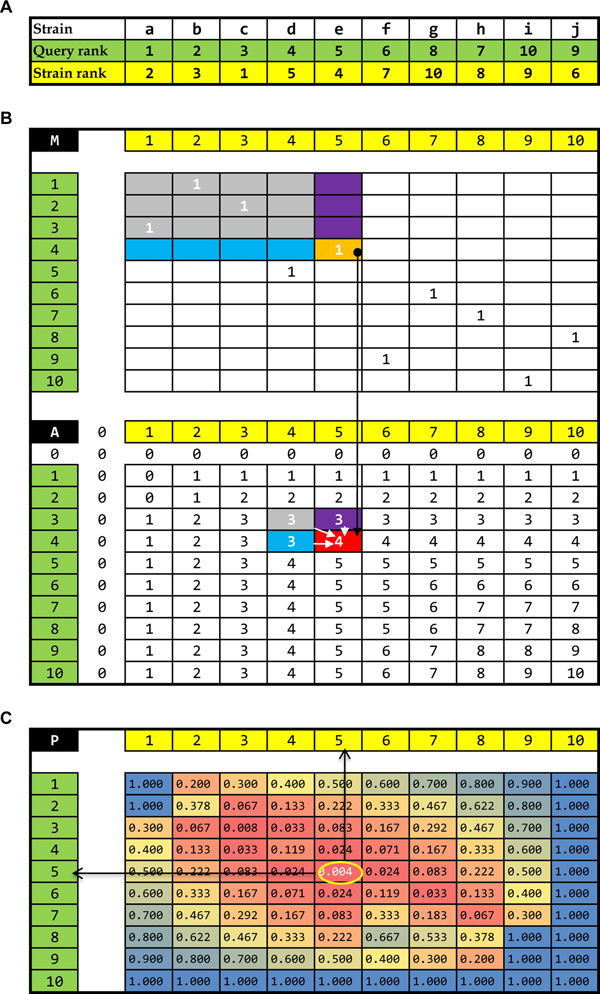
**Toy example showing how the rank-cutoff optimizer works**. (A) Ranks of each strain in virtual two query and target yeast fitness profiles to be compared are supposed to be deposited in Fit-RankDB. These profiles are also supposed to be generated using a virtual yeast deletion library comprising strain a to j. (B) Efficient calculation of a match number (or an overlapped strain number) accumulated under all possible rank-cutoffs of the query and the target by Dynamic programming (see the details in the Methods). For this calculation, first, rank matches of each strain should be expressed as the match matrix (M). In the M matrix, its row represents 'ranks in the query', its column 'ranks in the target', and its value 'the strain number with same rank in the query and the target'. Then, the current accumulated match number (in red-colored cell in the A matrix) is calculated by adding the current match number (in the orange-colored cell in the M matrix) to the previous accumulated match number (sky-colored cell plus purple-colored cell minus gray-colored cell in the A matrix). In this way, the accumulated match numbers regarding to all possible rank-cutoffs are efficiently calculated and stored in the A matrix. (C) The matrix of cumulative hyper-geometric p-values (P) is filled by calculating the equation (2) as the objective function (Hp) regarding to all possible rank-cutoffs, and used to find the rank-cutoffs with the minimized p-value as described in the equation (3), called optimal rank-cutoffs. The A matrix provides all of the parameters needed for equations (2) and (3) as follows: Its values represent the overlapped strain number in the equation (2); its row-names, the query strain number; its column-names, the target strain number in their respective rank-cutoffs; and its column or row length, the size of population. When the maximal rank-cutoff is set to 10 in the toy example, the query rank-cutoff 5 and the target rank-cutoff 5 shows the minimal p-value, 0.004. At those optimal rank-cutoffs, overlapping significance (hyper-geometric p-value) and overlapping score (Tanimoto coefficients) can be expressed as the similarity between the query and the target.

We will explain the rank cutoff optimizer step by step with a toy example. Suppose that we have two fitness profiles, one as a query and the other as a target. The first step is to convert the fitness values of each strain into their ranks as shown in Figure 2A. After each rank in the query and the target is sorted by their corresponding strain names as shown in Figure 2A, the rank matches of each strain can be expressed as a "match matrix" (**M**) as shown in the upper panel of Figure 2B. In the **M**, the rows represent the ranks in the query, and columns represent the ranks in the target, and the value indicates the strain number with the same rank in both the query and the target.

**Step 2. Constructing an accumulated match matrix (A): **Efficient calculation of the match number accumulated under all possible rank-cutoffs of the query and the target can be achieved by dynamic programming as follows:

(6) $$A_{ij}=M_{ij}+A_{ij-1}+A_{i-1j}-A_{i-1j-1}$$

In the equation (6), *M_ij _*is the match number in the rank *i *of the query and the rank *j *of the target, and *A_ij _*is the match number accumulated under the rank-cutoff *i *of the query and the rank-cutoff *j *of the target, which is schematically depicted in Figure 2B. This accumulated match matrix (**A**) provides the objective function, *Hp*, with all possible parameters for optimization. In **A**, values represent *m_ij _*as the overlapped strain number in the equation (2), its row-names *q_i _*indicate the query strain number and column-names *t_j _*indicate the target strain number in their respective rank-cutoffs *i *and *j*, and the column or row length *n *represents the size of the population. In the toy example of Figure 2, the maximal rank-cutoff *k *is set to the same as *n*.

**Step 3. Finding the optimal rank-cutoffs from a cumulative hyper-geometric *p-value *matrix (P): **The cumulative hyper-geometric *p-values *are calculated through the equation (2) of the objective function, *Hp *for all possible rank-cutoffs, stored in the **P**, and then used to find the rank-cutoffs with the minimized *p-value *as described in the equation (1). In the toy example of Figure 2, the query rank-cutoff of 5 and the target rank-cutoff of 5 show the minimal *p-value, 0.004 *(Figure 2C). This means that the best significant relationship between the query and the target in terms of overlap is observed at those rank-cutoffs, which are termed optimal rank-cutoffs in our study.

After this optimization, we can define the similarity between the query and the target at their optimal rank-cutoffs. In our study, we use two values: one is the minimized *p-value *as an overlapping significance, and the other is the Tanimoto coefficient as an overlapping score.

### Web application as the user interface

To search the similarities with all the profiles deposited in the FitRankDB using the rank-cutoff optimizer against a query profile, we provide users with two web frontends (Table 2). This web application uses a python-based Django framework and the rank-cutoff optimizer as the third party program implemented by C program. Significant top search results can be differently interpreted according to the types of query and target. Some clear interpretations are shown in Table 3.

**Table 2:** Available frontends in FitSearch web site.

Option	Description
FitSearchp	Search pre-compiled fitness rank database (FitRankDB) with a fitness profile of user.
FitSearchd	Search FitRankDB with the profile specified in FitRankDB.

There are more details in 'help' page in the web site.

**Table 3:** Biological interpretation about similarity between two fitness profiles

Query fitness profile	Target fitness profile	Biological interpretation of similar target treatment
**Chemical**-genetic (Hom)	**Chemical**-genetic (Hom)	Chemical effect (i.e. drug toxicity) with similar mode-of-action
**Chemical**-genetic (Het)	**Chemical**-genetic (Het)	Chemical effect with similar mode-of-action; Finding common direct drug target protein
**Chemical**-genetic (Hom)	**Genetic**-genetic (Hom)	Genetic effect (i.e. knock-out and mutations) on direct drug target protein gene
**Chemical/genetic**-genetic (Hom/Het)	**Biological** **functional annotation**: -Gene ontology -Protein complexes	Biological functions related to chemical or genetic effect

## Results

In FitSearch, we consider the following two design principles, universality and simplicity (See details in Methods). In brief, we adopt the use of rank statistics to compare two fitness profiles. This implies that any type of fitness scores can be universally converted into their corresponding ranks. The similarity of the two rank-transformed profiles can be easily calculated by rank-based comparison methods if we consider all of the profiles. In most situations, however, except for a relatively small number of top-ranked strains that are severely affected by the given drug, most strains can be considered as producing noise signals. Therefore, removal of such non-informative strains is necessary in order to calculate more accurate similarity measures and to make more meaningful comparison between profiles that may have been generated from different experimental treatments. An important question then arises: how should we define informative or non-informative strains? In other words, how should we set the optimal rank cutoffs for the two given profiles? The rank cutoff is an arbitrary value, can vary depending on viewpoint of the researcher, and is difficult to choose in advance for a pair of profiles.

**Figure 3:**
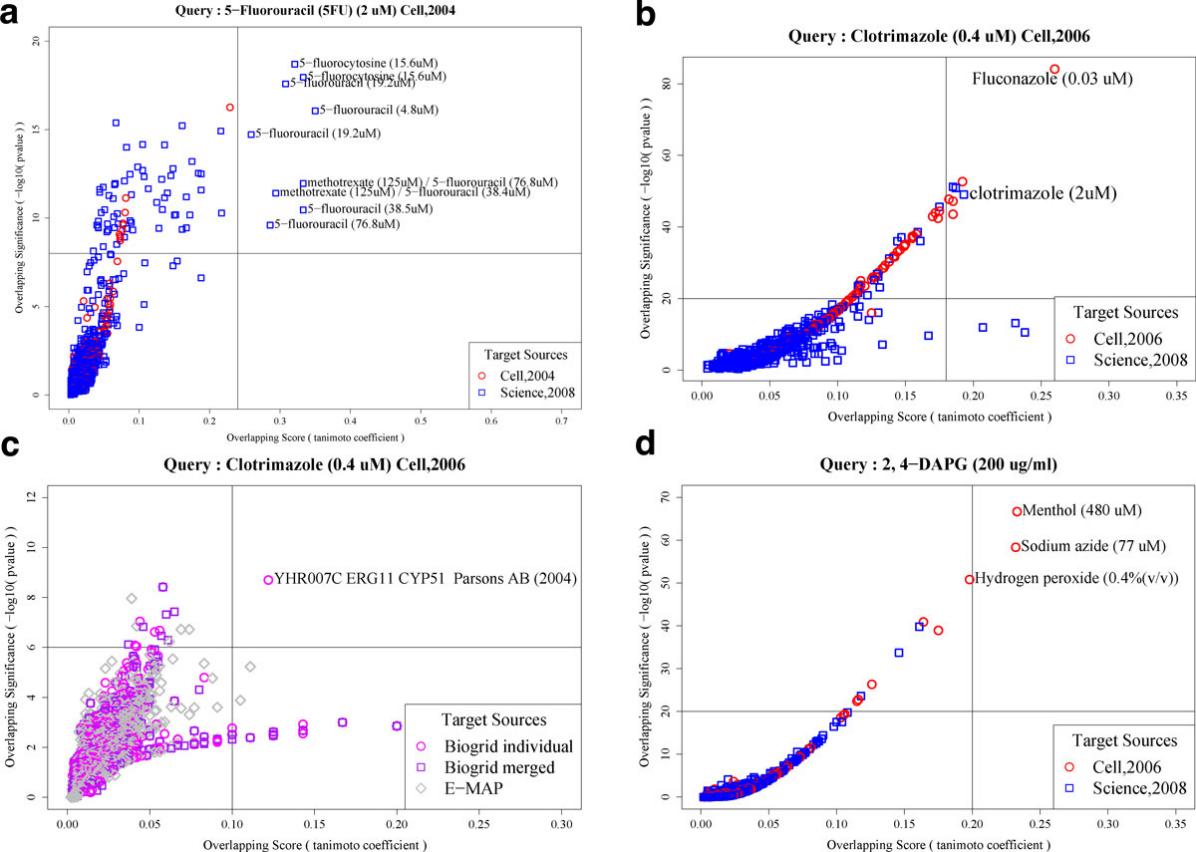
**Plot of an overlapping score and an overlapping significance as two-way cutoffs to show the most similar chemical or genetic effects to a query's effect**. (A) Two-way cutoff plot of the most similar chemical effects to the 5-Fluorouracil's effect. (B) Two-way cutoff plot of similar chemical effects to clotrimazole's effect. (C) Two-way cutoff plot of the most similar genetic effects to clotrimazole's effect. (D) Two-way cutoff plot of the most similar chemical effects to DAPG's effect. Target sources mean public chemical-genetic or genetic-genetic yeast profiles.

We solved this problem using an optimization method. Among all possible combinations of rank-cutoff values for a pair of profiles, FitSearch finds the optimal rank cutoffs by minimizing the p-value for the co-occurrence of the same set of strains in the two lists by chance, and finally returns two types of scores at those optimal rank cutoffs, viz., the Tanimoto coefficient and the p-value (Figure 2). These two scores based on overlapping strains efficiently display the similarity between profiles (Figure 3). Researchers do not need to define the cutoff values, which are automatically calculated by an internal optimizer in FitSearch.

To investigate whether FitSearch correctly identified fitness profiles sharing a similar mode-of-action, we performed a series of case studies, and validated the results by using prior knowledge and by performing experiments.

### Case study 1: comparing chemical-genetic profiles from different measurement platforms

Several high-throughput fitness profile datasets generated by using different platforms were deposited in FitRankDB. For example, the compendium named 'Cell, 2004' [4] contains heterozygous fitness profiles from the Merck company and fitDB collection [8], and 'Cell, 2006' contains homozygous fitness profiles from the Boone group [7]. In addition, the compendium named 'Science, 2008' [8] contains both homozygous and heterozygous fitness profiles from the fitDB collection. In this case study, we tested whether our method successfully identified the correct relationships between the chemical-genetic profiles generated from different measurement platforms.

Clustering the drugs according to the similarity between their corresponding chemical-genetic profiles provides insights into the unknown modes-of-action of drugs. This has been well demonstrated in 'Cell, 2006' where fitness profile data were generated from the platform using genome-wide homozygous deletion strains (i.e. a haploid collection). To generalize such an approach, we developed a method for measuring the similarity between chemical-genetic profiles generated by various types of measurement platforms (see Methods for details). We tested our new method using the chemical-genetic profiles of clotrimazole, latrunculin B, beomyl and cisplatin in 'Cell, 2006' as a query, and searched the combined database of 'Science, 2008' and 'Cell, 2006' which contain roughly 1,000 profiles. Under appropriate two-way cutoffs of overlapping score and significance, the top-ranked target profiles were either fitness profiles of the query drugs from different platforms or those of chemicals known to have a common mode-of-action in common with the query (Additional file 1). For example, the best hits for clotrimazole (0.4 μM) in 'Cell, 2006' are clotrimazole with a different dose (2 μM) in 'Science, 2008' and fluconazole (0.03 μM) in 'Cell, 2006' (Figure 3B). In addition, most of the target profiles of azoles from different platforms are listed in the top ranks.

In the case of genome-wide heterozygous deletion strains (i.e. a diploid collection), the main application of the chemical-genetic profiles is to find direct drug target protein candidates by screening drug-induced haploinsufficient strains [4,5]. Grouping of similar chemical-genetic profiles is not a typical approach, but was introduced to discover common targets and associated cellular functions for multiple classes of drugs [5,18]. To investigate the potential of FitSearch in a diploid collection, we selected as a query the yeast fitness profile of 5-flourouracil (5-FU), one of chemical-genetic profiles reported in 'Cell, 2004' which was the first compendium of chemical-genetic profiles based on a diploid collection. Even though this compendium was generated using roughly half of the genome-wide deletion strains, this study demonstrated that it was possible to identify the direct target protein of the drug and reported a novel target of 5-FU. We searched FitRankDB with 5-FU as a query using the FitSearchd program (Table 2). As expected, fitness profiles of 5-FU with different doses and generated from different platforms resided in the top ranks (Figure 3A). Additionally, similar chemicals, such as 5-fluorodeoxyuridine and 5-fluorocytosine showed high similarities. A synergistic effect of methotrexate and 5-fluorouracil was also detected by the similarity search.

### Case study 2: comparing chemical-genetic profiles and genetic-genetic profiles

In principle, deletion of a gene that encodes the target of an inhibitory compound should cause cellular effects that are similar to the inhibition of the target by drug treatment. In a proof-of-principle experiment, it was shown that for five different chemicals the chemical-genetic profiles were highly similar to the genetic interaction profiles of the target gene or genes in the target pathway [19]. To generalize this approach, we created a comprehensive compendium of global genetic interaction profiles by combining the interaction data from Biogrid [20] and E-MAP. Biogrid was deposited into FitRankDB in the forms of 'Biogrid individual' and 'Biogrid merged' (see Method for details).

For testing, we used the four chemical-genetic profiles of clotrimazole, latrunculin B, beomyl and cisplatin in 'Cell, 2006' as a query, and searched 'Biogrid individual', 'Biogrid merged' and 'E-MAP' to find the target profiles. We found that the top-ranked target profiles were relevant to the known mode-of-action of the query chemicals (Additional file 1). In particular, the target profile of ERG11 was detected at the first rank (Figure 3C); ERG 11 is known to be a direct target protein of clotrimazole.

### Case study 3: experimental validation

FitSearch is most beneficial to researchers who have performed a small-scale experiment using a yeast deletion collection. In a small-scale experiment, researchers usually generate one or two chemical-genetic profiles by colony- or OD-based platforms. That makes it difficult to perform data mining from public resources that were typically generated by chip-based platforms. To investigate how useful FitSearch is for a small-scale experiment, it was applied to obtain a hypothesis on the toxicity and mechanism of action of 2,4-diacetylphloroglucinol (2,4-DAPG). 2,4-DAPG is an antibiotic produced by *Pseudomonas fluorescens *that plays a key role in the ability of the bacterium to suppress phytopathogenic fungi. 2,4-DAPG has broad antibiotic activity, affecting organisms ranging from bacteria to higher plants. The biosynthesis and regulation of 2,4-DAPG in *P. fluorescens *has been well described. However, the mode of action of the antibiotic against target fungi has not been described in detail.

For testing, we selected common mutants in a haploid collection that showed significant growth defects in colony- and OD-based screening when exposed to DAPG (manuscript in preparation), and transformed them into binary query profiles. Public chemical-genetic profiles from 'Cell, 2006' and 'Science, 2008' were used as the target sources for a similar mode-of-action drug search and public genetic-interaction datasets of 'Biogrid individual', 'Biogrid merged', and 'E-MAP' were used as the target sources to find the direct drug target candidates. Under appropriate two-way cutoffs of overlapping score and significance, (1R, 2S, 5R)-2-isopropyl-5-methylcyclohexanol (menthol), sodium azide, and hydrogen peroxide were found to have similar toxicity and mechanism of action to 2,4-DAPG (Figure 3D and Additional file 1). However, no genetic effect similar to 2,4-DAPG chemical effect was detected.

### Predicting gene-gene interactions

A genetic interaction can be defined as a synergetic phenotype that cannot be elucidated by simply combining the phenotypes of distinct gene perturbations. A typical way to detect genetic interaction is to make use of synthetic lethal genetic screens and synthetic dosage lethal screens. However, due to the vast number of gene-gene combinations, genome-wide screening had not been possible until the Synthetic Genetic Array (SGA) method [21] became available. Recently, genome-scale SGA analysis was used to chart the genetic interaction map in budding yeast [22]. The profiles of quantitative scores of genes in SGA screen were stored in FitRankDB. In addition, we tested whether our FitSearch algorithm could reveal genetic interactions. We carried out FitSearchd (Table 2) for all genetic fitness profiles in FitRankDB first and predicted genetic interactions if the two genetic profiles had a significant p-value and Tanimoto coefficient (p-value < 1e-10, Tc > 0.1). About 12% of interactions predicted in this study are real genetic interactions according to the work by Costanzo *et al*. [22]. All other interactions would be either false positives or novel genetic interactions not previously detected. In Table 4, we compiled the top-10 list of these interactions sorted by Tanimoto coefficient. Although the results we obtained are not included in previous SGA screening work, there is clear evidence supporting that these predicted interactions are not false positives. These results indicate that FitSearch can find new genetic interactions, which cannot be identified by SGA analysis and that these two techniques can complement each other.

**Table 4:** FitSearch can detect genetic interactions that cannot be detected by SGA analysis.

Rank	Gene1	Gene2	Tc	P-value	Note
1	YPL022W RAD1	YML095C RAD10	1	3.44E-29	Single-stranded DNA endonucleases (with each other)
2	YDL040C NAT1	YHR013C ARD1	0.94	1.18E-317	Subunit of the N-terminal acetyltransferase NatA (Nat1p, Ard1p, Nat5p)
3	YCR009C RVS161	YDR388W RVS167	0.91	3.37E-114	Manually curated by [27]
4	YPL020C ULP1	YKR082W NUP133	0.91	3.79E-28	Overexpression of ULP1 rescues a nup133 rad27 or nup60 rad27 double mutant [28]
5	YJL194W CDC6	YHR118C ORC6	0.91	1.42E-72	ORC6-rxl and chromosomal deletion of the Cdc6 leads to slow growth phenotype [29]
6	YMR125W STO1	YPL178W CBC2	0.88	2.62E-40	Both are subunits of cap-binding protein complex
7	YMR224C MRE11	YNL250W RAD50	0.88	1.34E-171	MRE11 is a subunit of a complex with Rad50p and Xrs2p
8	YBR175W SWD3	YAR003W SWD1	0.88	4.12E-54	Both are subunits of the COMPASS (Set1C) complex
9	YDR166C SEC5	YLR166C SEC10	0.86	2.30E-32	Both are subunits of the exocyst complex
10	YNL041C COG6	YNL051W COG5	0.8	1.56E-96	Both are components of the conserved oligomeric Golgi complex

The table shows the top 10 results that are not included in the genetic interaction list by SGA analysis [22]. Notes without references are retrieved from the Saccharomyces Genome Database (SGD) [26]. P-values, here, are corrected considering multiple tests.

## Discussion

Yeast deletion collections have been successfully used to infer modes-of-actions of drugs especially by profiling chip-based chemical-genetic and genetic-genetic interactions on a genome-wide scale [4,5,7,9]. In addition, tens of thousands of those profiles are publicly available. However, if researchers only have a few yeast profiles to their drugs of interest with unknown toxicity mechanisms, it is not easy to compare them with public resources to investigate whether similar profiles exist. One of the main reasons is that, typically, the chemical-genetic profiles have been generated on non-chip based measurement platforms, such as a simple 96-well spotting assay or high-density colony measurement on an agar plate [10], high-density well plate-based optical density [11], or fluorescence [12] measurements in a liquid culture. Furthermore, different fitness scoring methods are also problematic. Therefore, there is an urgent need for developing platform- and scoring method-independent ways to compare fitness profiles for more efficient utilization of the public resources.

In previous studies, the conventional similarity measures, such as the Pearson correlation coefficient, were typically used for comparing fitness profiles. However, such simple similarity measures can be applied only to the profiles generated by well-controlled experiments using a single measurement platform. The probability of chance overlap by chance between two profiles was also used to measure their similarity, but was only applicable to fitness profiles with pre-defined significant thresholds [19]. Such thresholds are typically arbitrary and vary depending on the experimental setup and the researcher's viewpoint. This requires a more universal and simple way to measure similarities.

Here, we demonstrated the general usability of FitSearch as a new similarity measure of yeast fitness profiles by literature-based and experimental case studies. In the first literature-based case study, we tested whether our new method was able to find drugs with similar modes-of-action even if their fitness profiles had been generated by different measurement platforms. A previous study showed that similarities of chemical-genetic profiles generated from the same platform tended to imply similar modes-of-action of drugs [7]. Our new similarity measure, FitSearch, can generalize such an approach even with chemical-genetic profiles obtained from different experimental platforms. For example, we showed that drugs very similar or the same as 5-FU and clotrimazole were detected in the top rank after FitSearch (Figure 3A and 3B).

In the second literature-based case study, we tested whether the new method could be applied to measure the similarity between a chemical-genetic profile and a genetic-interaction profile. In principle, deletion of a gene that encodes the target of an inhibitory compound should cause cellular effects that are similar to inhibition of the target by a drug treatment. This proof-of-principle experiment was successfully performed [19]. Our FitSearch is a generalization of this type of approach. We showed that the target protein of clotrimazole, ERG 11 was detected using chemical- and genetic-genetic profiles generated in different platforms (Figure 3C).

FitSearch benefits researchers performing small-scale experiments using yeast deletion collection because in these studies, only one or two chemical-genetic profiles are generated by colony- or OD-based platforms. This makes it difficult to perform data mining from public resources, as these data are typically generated on the chip-based platforms. An example is a recent study (manuscript in preparation) that generated yeast fitness profiles to 2,4-DAPG, an antibiotic with a poorly understood mode-of-action. Through similarity searching of FitRankDB using the web-frontend, FitSearchp (Table 2), we found three similar compounds; menthol, sodium azide and H_2_O_2 _in the top rank (Figure 3D). Menthol is known to cause a perturbation in the lipid fraction of the membrane, altered membrane permeability and consequential leakage of intracellular materials [23]. Sodium azide has been known as a rapid and reversible inhibitor of the cytochrome c oxidase-respiratory chain complex IV, through enhanced cytochrome c holoenzyme dissociation [24]. Membrane associated protein kinase C activity can also be altered by sodium azide [24]. Marino *et al.*[25] reported that sodium azide increases intracellular calcium in mammalian systems, causing azide neurotoxicity. Hydrogen peroxide (H_2_O_2_) can damage proteins, lipids, and DNA. The primary source of reactive oxygen species such as H_2_O_2 _is free-leakage of electrons, which is generated by the mitochondrial respiratory system. Based on the modes-of-action of these three similar drugs, it was suggested that mechanism of action of 2,4-DAPG may involve disturbing cell membrane permeability, triggering of a reactive oxygen burst, and interruption of cell homeostasis.

## Conclusions

Yeast deletion collections have been successfully used to infer mode-of-actions of drugs, in particular by profiling chip-based chemical-genetic and genetic-genetic interactions on a genome-wide scale. For optimal use of public resources, we designed FitRankDB as a general repository of fitness profiles, and developed FitSearch as a new similarity measure between such profiles. We showed that our new repository and algorithm are beneficial to researchers who are attempting to obtain hypothesis regarding the unknown modes-of-action of a bioactive compound through a small-scale experiment with yeast deletion collections from different platforms, specifically non-chip based platforms.

## Competing interests

The authors declare that there are no competing interests.

## Authors' contributions

ML, SH and DK conceived of the study. SH and HC designed an algorithm and implemented it. ML maintains the web service. YK and DW performed an experimental case study. ML, SH, YK and HC analyzed data. All authors wrote, read and approved the final manuscript.

## Declarations

The publication costs for this article were funded by the Korean Government, the Ministry of Education, Science & Technology (MEST) [2009-0086964].

This article has been published as part of *BMC Genomics *Volume 14 Supplement 1, 2013: Selected articles from the Eleventh Asia Pacific Bioinformatics Conference (APBC 2013): Genomics. The full contents of the supplement are available online at http://www.biomedcentral.com/bmcgenomics/supplements/14/S1.

## Supplementary Material

Additional file 1 **Summary of FitSeach results of Clotrimazole, Latrunculin B, Benomyl, Cisplatin, and 2,4-DAPG**. Click here for file
