# Supplementary material for: FitSearch: a robust way to interpret a yeast fitness profile in terms of drug's mode-of-action
Source: BMC Genomics. 2013 Jan 21;14(Suppl 1):S6. doi: 10.1186/1471-2164-14-S1-S6 (PMC3549813; doi:10.1186/1471-2164-14-S1-S6)
Supplement: Additional file 1 — Summary of FitSeach results of Clotrimazole, Latrunculin B, Benomyl, Cisplatin, and 2,4-DAPG. [file 1471-2164-14-S1-S6-S1.pdf]

Summary of FitSeach results of Clotrimazole, Latrunculin B, Benomyl, Cisplatin, and 2,4-DAPG

**Query profile**	Treatment	**Cutoff**		**Top-ranked profiles in Target Sources**		**Drug's mode-of-action**
		Overlapping Score	Overlapping Significance	Science, 2008 or Biogrid individual	Cell, 2006 or Biogrid merged	
[F1.16901791.3] Clotrimazole (0.4 nM) (Cell, 2006)	Chemical effect	> 0.18	< 1E-40	[F1.18420932.1.1.26] clotrimazole (2uM) [F1.18420932.1.2.28] miconazole (50nM)	[F1.16901791.81] Fluconazole (0.03 uM)	Clotrimazole and fluconazole, chemical analogs and antifungal agents that target Erg11 (ref), a protein encoded by an essential gene in the ergosterol biosynthesis pathway
	Genetic effect	> 0.12	< 1E-8	[F1.16381927.1.3383] YHR007C **ERG11** Parsons(2004)	No hit	
[F1.16901791.24] Latrunculin B (30 uM) (Cell,2006)	Chemical effect	> 0.18	< 1E-40	[F1.18420932.1.1.268] Latrunculin (0.78uM)	[F1.16901791.73] Cytochalasin A (21 uM)	Latrunculin B and cytochalasin A are known for actin binding agents; BNI1 encodes formin, nucleates the formation of linear actin filaments, involved in cell processes such as budding and mitotic spindle orientation which require the formation of polarized actin cables, functionally redundant with BNR1
	Genetic effect	> 0.12	< 1E-15	[F1.16381927.1.2967] YNL271C BNI1 Tong(2001) [F1.16381927.1.560] YNL271C BNI1 Tong(2004) [F1.16381927.1.4089] YOR326W MYO2 Tong(2004)	[F1.16381927.2.1217] YNL271C BNI1 [F1.16381927.2.858] YOR326W MYO2	
[F1.16901791.4] Benomyl (0.12 uM) (Cell,2006)	Chemical effect	> 0.15	< 1E-35	[F1.18420932.1.1.316] benomyl (10ug/ml)	[F1.16901791.64] Extract 95-57 (30 ug/ml)	Microtuble-depolymerizing agents;In *S.cerevisiae*, *RBL2* encodes the yeast structural and functional homolog of mouse beta-tubulin cofactor A.
	Genetic effect	> 0.12	< 1E-13	[F1.16381927.1.1933] YOR265W RBL2 Tong(2004)	[F1.16381927.2.211] YOR265W RBL2	

[F1.16901791.36]<br>Cisplatin (0.17 mM)<br>(Cell,2006)	Chemical effect	> 0.3	< 1E-20	[F1.18420932.1.2.320]<br>oxaliplatin (4000uM)<br>[F1.18420932.1.2.291]<br>cisplatin (500uM)<br>[F1.18420932.1.2.139]<br>cisplatin (250uM)<br>[F1.18420932.1.2.324]<br>psoralen irradiated (0.5uM)	[F1.16901791.76]<br>Camptothecin (30 uM)	Platinum-derivatives (i.e. cisplatin and oxalipatin) are alkylating-like DNA inter- and intra-cross linking agents as DNA replication inhibitor.; DIA2 is origin-binding F-box protein that forms an SCF ubiquitin ligase complex playing a role in DNA replication, involved in invasive and pseudohyphal growth; *RAD27* encodes a multi-functional nuclease involved in processing Okazaki fragments during DNA replication, base excision repair (BER), and maintaining genome stability
	Genetic effect	> 0.12	< 1E-13	No hit	[F1.16381927.2.1595]<br>YOR080W DIA2<br>[F1.16381927.2.2340]<br>YKL113C RAD27<br>[F1.16381927.2.1465]<br>YMR190C SGS1	
2,4-DAPG (200 ug/ml)	Chemical effect	> 0.20	< 1E-20	No hit	[F1.16901791.15]<br>Menthol (480 uM)<br>[F1.16901791.11]<br>Sodium azide (77 uM)<br>[F1.16901791.26]<br>Hydrogen peroxide (0.4%(v/v))	Unknown
	Genetic effect	> 0.1	< 1E-6	No hit	No hit	
